# Antisense down-regulation of the strawberry β-galactosidase gene *FaβGal4* increases cell wall galactose levels and reduces fruit softening

**DOI:** 10.1093/jxb/erv462

**Published:** 2015-11-19

**Authors:** Candelas Paniagua, Rosario Blanco-Portales, Marta Barceló-Muñoz, Juan A. García-Gago, Keith W. Waldron, Miguel A. Quesada, Juan Muñoz-Blanco, José A. Mercado

**Affiliations:** ^1^Instituto de Hortofruticultura Subtropical y Mediterránea “La Mayora” (IHSM-UMA-CSIC), Departamento de Biología Vegetal, Universidad de Málaga, 29071 Málaga, Spain; ^2^Departamento de Bioquímica y Biología Molecular, Universidad de Córdoba, 14071 Córdoba, Spain; ^3^IFAPA, Centro de Churriana, 29140 Málaga, Spain; ^4^Institute of Food Research, Norwich Research Park, Colney, Norwich, NR4 7UA, UK; ^5^Departamento de Biología Vegetal, Universidad de Málaga, 29071 Málaga, Spain

**Keywords:** β-galactosidase, cell wall, *Fragaria × ananassa*, fruit ripening, fruit softening, pectins.

## Abstract

Strawberry softening is characterized by an increase in the solubilization and depolymerization of pectins from cell walls. Galactose release from pectin side chains by β-galactosidase enzymes has been proposed as one reason for the increase in soluble pectins. A putative β-galactosidase gene, *FaβGal4*, has been identified using a custom-made oligonucleotide-based strawberry microarray platform. *FaβGal4* was expressed mainly in the receptacle during fruit ripening, and was positively regulated by abscisic acid and negatively regulated by auxins. To ascertain the role of *FaβGal4* in strawberry softening, transgenic plants containing an antisense sequence of this gene under the control of the CaMV35S promoter were generated. Phenotypic analyses were carried out in transgenic plants during three consecutive growing seasons, using non-transformed plants as control. Two out of nine independent transgenic lines yielded fruits that were 30% firmer than control at the ripe stage. *FaβGal4* mRNA levels were reduced by 70% in ripe fruits from these selected transgenic lines, but they also showed significant silencing of *FaβGal1*, although the genes did not share significant similarity. These two transgenic lines also showed an increase in pectin covalently bound to the cell wall, extracted using Na_2_CO_3_. The amount of galactose in cell walls from transgenic fruits was 30% higher than in control; notably, the galactose increase was larger in the 1 M KOH fraction, which is enriched in hemicellulose. These results suggest that *FaβGal4* participates in the solubilization of covalently bound pectins during ripening, reducing strawberry fruit firmness.

## Highlight

We identified a strawberry β-galactosidase gene involved in fruit ripening. Its silencing increases cell wall galactose levels and fruit firmness, suggesting galactose metabolism has a key role in strawberry softening.

## Introduction

Softening is one of the most characteristic aspects of the fruit ripening process. In soft fruits, such as strawberry, the soft melting texture is highly appreciated by consumers, but it poses a major problem for strawberry producers, determining the short postharvest shelf life of this fruit and limiting its storage and postharvest transport. During ripening, fruit cell walls are extensively modified, and these changes are the most important factor leading to fruit softening. In general, cell wall modifications that accompany softening involve the solubilization of pectin polymers, the depolymerization of pectins and matrix glycans, and the loss of neutral sugars from pectin side chains (Brummell, 2006; Goulao and Oliveira, 2008; Mercado *et al.*, 2011). All these processes often occur concurrently during fruit ripening, although the extension of these modifications greatly depends on the kind of fruit (Brummell, 2006; Mercado *et al.*, 2011).

Strawberry softening is characterized by a moderate increase in pectin solubilization—i.e. an increase in the amount of pectins loosely bound to the cell wall—and depolymerization (Posé *et al.*, 2011). Functional studies of genes encoding pectinase enzymes, such as polygalacturonase (Quesada *et al.*, 2009) or pectate lyase (Jiménez-Bermúdez *et al.*, 2002; Youssef *et al.*, 2009; Youssef *et al.*, 2013), support a key role of pectin disassembly in strawberry softening. Transgenic fruits with low expression levels of these genes were significantly firmer than control fruits and displayed a reduction in solubilization and depolymerization of polyuronides (Santiago-Doménech *et al.*, 2008; Posé *et al.*, 2013; Posé *et al.*, 2015). The molecular mechanism underlying pectin disassembly is unclear (Paniagua *et al.*, 2014). Many fruits, including strawberry, show a loss of neutral sugars, mainly arabinose and galactose, during ripening (Gross and Sams, 1984; Redgwell *et al.*, 1997). The removal of these carbohydrates from rhamnogalacturonan I (RG-I) polyuronides by β-galactosidases (β-Gal) and α-arabinofuranosidases has been suggested as one of the possible reasons for the increase of soluble pectins (Koh and Melton, 2002; Brummell, 2006).

β-galactosidases (EC 3.2.1.23) are glycosyl hydrolases characterized by their ability to hydrolyse terminal, non-reducing β-D-galactosyl residues from numerous β-D-galactoside substrates (Pérez-Almeida and Carpita, 2006; Tateishi, 2008). In most fruits, β-galactosidases are encoded by small gene families with different patterns of expression that could play distinct roles in fruit development (Smith and Gross, 2000; Mwaniki *et al.*, 2005; Tateishi *et al.*, 2007; Othman *et al.* 2011). Functional analyses of β-galactosidases genes using transgenic plants have only been carried out in the climacteric model fruit tomato (*Solanum lycopersicum*) with contrasting results. Antisense *TBG4* tomato fruits displayed reduced *TBG4* mRNA levels and free cell wall galactose only at the onset of ripening, but softening of ripe fruits decreased by 40% (Smith *et al.*, 2002). By contrast, neither the silencing of *TBG1* (de Silva and Verhoeyen, 1998) nor *TBG3* (Carey *et al.*, 2001) modified tomato fruit firmness. In *TBG6* antisense tomato plants, fruit firmness was also similar to that of control plants, but these fruits showed structural alterations in the cuticle that increased fruit cracking (Moctezuma *et al.*, 2003).

In strawberry (*Fragaria × ananassa*), a non-climacteric fruit, β-galactosidase activity increases during fruit development and remains high in ripe fruit (Trainotti *et al.*, 2001; Figueroa *et al.*, 2010). At the molecular level, Trainotti *et al.* (2001) isolated three full-length cDNAs encoding β-galactosidase genes *FaβGal1* to *FaβGal3*. Although all of them could be detected in fruit and vegetative tissues, only *FaβGal1* showed an expression pattern that could be related to the fruit ripening process, with the other two genes expressed mainly in green, immature fruits. Transcriptomic studies performed in our research group have identified a large group of genes whose expression increases throughout strawberry fruit ripening. One of these genes, *FaβGal4* (accession number KR189030), displays significant sequence homology with putative β-galactosidase from higher plants. The main goal of this study was the functional characterization of this gene. For this purpose, transgenic strawberry plants carrying an antisense sequence of *FaβGal4* were generated and the effects of *FaβGal4* down-regulation in fruit firmness and cell wall structure were analysed.

## Materials and methods

### Plant material

Strawberry plants (*Fragaria* × *ananassa* Duch., cv. Camarosa) were grown under field conditions in Huelva (south-west Spain). Fruits were harvested at different developmental stages: small-sized green fruits (G1, 2–3g), medium-sized green fruits (G2, 3–5g), full-sized green fruits (G3, 4–7g), white fruits (W, 5–8g), and full-ripe red fruits (R, 6–10g). Vegetative tissues, such as runners, flowers, and expanding leaves, were also harvested. For the genetic transformation, *in vitro* micropropagated plants, cv. Chandler, were used. All tissues and fruit samples were immediately frozen in liquid nitrogen and stored at −80°C.

### *Cloning and sequence analysis of full-length cDNA of* FaβGal4

The full-length cDNA corresponding to the *FaβGal4* gene was isolated from a *Fragaria* × *ananassa* R stage fruit cDNA library (Medina-Escobar *et al.*, 1997). The deduced amino acid sequence and the phylogenetic tree construction were performed using the Lasergene software package (DNASTAR).

### Auxin and nordihydroguaiaretic acid treatments

Achenes of two sets of 50 middle-sized green fruits (G2) each, still attached to the plant, were carefully removed using the tip of a scalpel blade. One set of deachened G2 fruits was treated with the synthetic auxin 1-naphthalenacetic acid (NAA) in lanolin paste (1mL) with 1mM NAA in 1% (w/v) DMSO. The other set of G2 deachened fruits (control group) were treated with the same paste, but without NAA. Both treatments were applied over the whole fruit surface. All fruits were harvested 5 days after treatment, immediately frozen in liquid nitrogen, and stored at −80°C. During the course of the assays, the fruits reached the G3–W developmental stages.

Nordihydroguaiaretic acid (NDGA) is an ideal inhibitor of the 9-cis-epoxycarotenoid dioxygenase enzyme and was used to block abscisic acid (ABA) biosynthesis (Creelman *et al.*, 1992). The lowest NDGA concentration that completely blocks ABA accumulation is 100 μM, as determined by preliminary tests in tomato fruit (Zhang *et al.*, 2009). Strawberry fruits (*Fragaria* × *ananassa*, cv. Elsanta) were used at the mature G–W stages for the purposes of this study. All fruits (ten fruits per treatment) were carefully injected with 1–2mL of NDGA (100μM) sterile solution or sterile water (control fruits) using a hypodermic syringe. Three replications were conducted for each treatment. The samples were harvested after 8 days of treatment, when the fruits reached the R developmental stage, then frozen in liquid nitrogen and stored at −80°C. Fruits treated with NDGA were white, whereas control fruits were red. These samples were used to determine relative expression of *FaβGal4*.

### RNA isolation

Total RNA was isolated from independent pools of strawberry fruits at different growth and ripening stages and from vegetative tissues, in accordance with Asif *et al.* (2000). Achenes were always removed from fruit and only receptacle RNA was extracted and puriﬁed. RNA obtained was treated with RNase-free DNase I (Invitrogen) and purified through the RNeasy Mini kit (Qiagen). RNA concentration and purity were evaluated using a NanodropTM spectrophotometer ND-1000 (Thermo Scientific) and by 1% agarose gel electrophoresis.

### Expression analysis by quantitative real-time PCR

Gene expression analysis of *FaβGal4* was performed by quantitative real-time (qRT)-PCR through an iCycler (BioRad) device, as previously described (Benítez-Burraco *et al.*, 2003). *FaβGal4* gene primer sequences for quantitative amplification were 5′- CAG CCA CCC ACT CCT CTA TAA CCA GTT -3′ and 5′- GCG AAG CAG TAA AAT ACG AAG CAA AGC-3′. Each reaction was performed at least in triplicate and the corresponding cycle threshold (C_t_) values were normalized using the C_t_ value corresponding to an *interspacer 26S-18S* strawberry RNA gene (housekeeping gene) (Benítez-Burraco *et al.*, 2003; Molina-Hidalgo *et al.*, 2013). All of these values were subsequently used to determine the relative increase or decrease in *FaβGal4* expression in the samples in comparison to that of the control gene in accordance with Pedersen (2001). *Interspacer 26S-18S* (primers: 5′-ACC GTT GAT TCG CAC AAT TGG TCA TCG-3′ and 5′-TAC TGC GGG TCG GCA ATC GGA CG-3′) was selected as the control gene owing to its constitutive expression throughout all of the different experimental conditions tested. The efficiency of each particular qRT-PCR and the melting curves of the products were also analysed to ensure the existence of a single amplification peak corresponding to a unique molecular species. The expression levels of the different β-galactosidase genes in control and transgenic antisense *FaβGal4* fruits were measured by qRT-PCR as described above, using the following primers: *FaβGal1* 5′-AAA GGC AAG CAG GAC ATA CC-3′ and 5′-CCA TAA CAT CAG CCC AAA CC-3′; *FaβGal2* 5′-TTC ATG GCT CTC CTC TGC TT-3′ and 5′-ACA TCC AAG CCT CCA TCT T-3′; *FaβGal3* 5′-TTC ATG GCT CTC CTC TGC TT-3′ and 5′-ACA TCC AAG CCT CCA TCT T-3′; *FaβGal4* 5′-GAT GCT TCT CGG TAT CC-3′ and 5′-TGT AAT CGC TTC TTC TGT TCC T-3′.

### *Binary vector for antisense* FaβGal4 *silencing and generation of transgenic strawberry plants*

A 300-bp non-conserved region of the *FaβGal4* gene in antisense orientation was cloned into the pK7WG2 binary vector using Gateway technology (Invitrogen, Darmstadt, Germany) for antisense silencing of *FaβGal4*. The forward primer 5′-AGA GGA GAT GCT CGG TCT CGG TAT C-3′ and reverse primer 5′-TGG CAT AGC GCT TAA ATA GTT CAT TCA GTT-3′ were used. The resulting fragment was cloned into pCR8/GW/TOPO (Invitrogen) and then transferred to the Gateway pK7WG2 vector by way of a specific recombination of both plasmids using LR clonase (Invitrogen). The resulting plasmid (pK7WG2-*FaβGal*) was tested through sequencing and restriction analyses prior to strawberry plant transformation. The plasmid was introduced into *Agrobacterium tumefaciens* strain AGL1 by electroporation.

Leaf discs of strawberry plants, cv. Chandler, micropropagated *in vitro* were used as explants for *Agrobacterium*-mediated transformation experiments, as described in Barceló *et al.* (1998). Explants were inoculated with a diluted culture of *A. tumefaciens* and selected in 25mg L^−1^ kanamycin. After 7–8 months of selection, kanamycin-resistant shoots were acclimated and transferred to the greenhouse. Transgenic mother plants were propagated by runners and the daughter plants were used for phenotypic analysis. The presence of the transgenes in these putative transgenic lines was confirmed by PCR amplification of a 220-bp fragment belonging to the *nptII* gene.

### Phenotypic analysis of transgenic plants

Transgenic plants were evaluated during three consecutive growing seasons, using non-transformed plants, cv. Chandler, as control. Plants were grown in a greenhouse under natural temperature and light conditions and fruit was collected from March to July. During the first year, nine independent antisense *FaβGal4* lines were analysed. Eight plants per line and a minimum of 10 ripe fruits per line were evaluated. During the second and third year, two selected lines showing higher fruit firmness than control were evaluated. Thirty plants per line and 50–100 ripe fruits per line were analysed in both years. Fruits were harvested at the stage of full ripeness, when the fruit surface was completely red, and the weight, size, colour, soluble solids, and firmness were recorded. Colour was measured using a colorimeter (Minolta Chroma Metre CR-400, Osaka, Japan). The instrument was calibrated with a standard white and a standard black reflective plate before use. The L* a* b* colour space parameters (lightness, redness, yellowness) were recorded. Soluble solids were measured using a refractometer (Atago N1), and firmness using a hand penetrometer (Effegi) with a cylindrical needle of 9.62mm^2^ area. 

### DNA extraction and enzyme assays

Genomic DNA was extracted from young strawberry leaves using Qiagen DNeasy Plant kit. Previously, plant material had been washed three times with washing buffer solution consisting of 100mM sodium acetate buffer (pH 5), 20mM EDTA, 0.2M sorbitol, 2% polyvinylpyrrolidone (PVP, molecular weight 40 000), and 1% β-mercaptoethanol (Mercado *et al.*, 1999).

β-galactosidase activity was measured in ripe fruits according to Figueroa *et al.* (2010). Protein extraction was carried out by grinding 10g of strawberry fruits under liquid nitrogen into a fine powder. The powder was homogenized using an Ultra-Turrax (Janke & Kunkel) with 20mL of cold extraction buffer [0.05M Na-acetate buffer, pH 6, 1% (w/v) polyvinylpolypyrrolidone (PVPP)] containing 1 M NaCl. Homogenates were stirred at 4°C for 3h and then centrifuged at 11 000 *g* for 30min. The supernatant was used to determine β-galactosidase activity by using p-nitrophenyl-β-D-galactopyranoside (Sigma) as the substrate. To measure exo-galactanase activity, fruit tissue was extracted as described by Trainotti *et al.* (2001). Briefly, the powder was homogenized with cold extraction buffer, centrifuged at 11 000 *g* for 15min, and the pellet washed with 10ml of extraction buffer without PVPP. After centrifugation, the pellet was resuspended in 0.05M Na-acetate buffer, pH 6, containing 1 M NaCl and stirred for 5 h at 4°C. After extraction, the insoluble material was removed by centrifugation and the supernatant was dialyzed against 0.05M Na-acetate buffer, pH 4.5. A lupin galactan pretreated with α-L-arabinofuranosidase (Megazyme) was used as the substrate for exo-galactanase activity as described by Carey *et al.* (1995). The galactosidase released in the assay was estimated by measuring the release of reducing sugars using 2-cyanoacetamide (Gross, 1982). Protein extractions were performed in triplicate.

### Cell wall analysis

The cell walls were extracted from frozen ripe fruits following the method of Redgwell *et al.* (1992) with some modifications as previously described by Santiago-Doménech *et al.* (2008). Briefly, 20 g of fruit were homogenized in a 40ml mix of phenol, acetic acid, and water (PAW, 2:1:1, w:v:v). The homogenate was centrifuged at 4000 *g* for 15min and the supernatant filtered through Miracloth (PAW fraction). The pellet was treated overnight with 90% DMSO to solubilize the starch. The extract was then centrifuged at 4000 *g* and the pellet washed twice with distilled water. The water fraction was discarded and the pellet, the cell wall material (CWM), was lyophilized and weighed. After extraction, the CWM was sequentially fractionated following the procedure of Santiago-Doménech *et al.* (2008). Briefly, 300mg of the CWM was sequentially extracted overnight with deionized water, 0.05M *trans*-1,2-diaminocyclohexane-*N*,*N*,*N′N′*-tetraacetic acid (CDTA) in 0.05M acetate buffer (pH 6), 0.1 M Na_2_CO_3_ containing 0.02 M NaBH_4_, 1 M KOH containing 0.02M NaBH_4_, and 4 M KOH containing 0.02M NaBH_4_. All fractions were extensively dialyzed against pure water. Then, the extracts were concentrated with a rotary evaporator and freeze-dried. Three independent fractionations per CWM sample were performed.

The uronic acid (UA) content was measured using the carbazole method (Filisetti-Cozzi and Carpita, 1991) using galacturonic acid as the standard. For neutral sugar analysis, the CWM and the different cell wall fractions were hydrolysed with 72% (w/w) sulphuric acid. After neutralization with ammonia, carbohydrates were derivatized to alditol acetates and analysed by gas chromatography with flame ionization detection (Blakeney *et al.*, 1983). Gas chromatography analysis was carried out in triplicate.

Infrared spectra were recorded on a Jasco FT/IR-4100 (Spain) spectrometer coupled to an attenuated total reflectance (ATR) accessory (MIRacle ATR, PIKE Technologies, USA). Spectra Manager v.2 software (Jasco, Spain) was used to correct for both ATR effect and atmospheric contributions from carbon dioxide and water vapour across the full spectral range.

### Statistical analysis

SPSS software was used for all the statistical analyses. Levene’s test for homogeneity of variances was performed prior to ANOVA, and Dunnett’s multiple comparison test or Tamhane’s T2 test was used for mean separation. A Student’s *t* test or Mann–Whitney U test was used for pair comparisons.

## Results

### *Sequence analysis of* FaβGal4 *gene and protein*

**Fig. 1.**
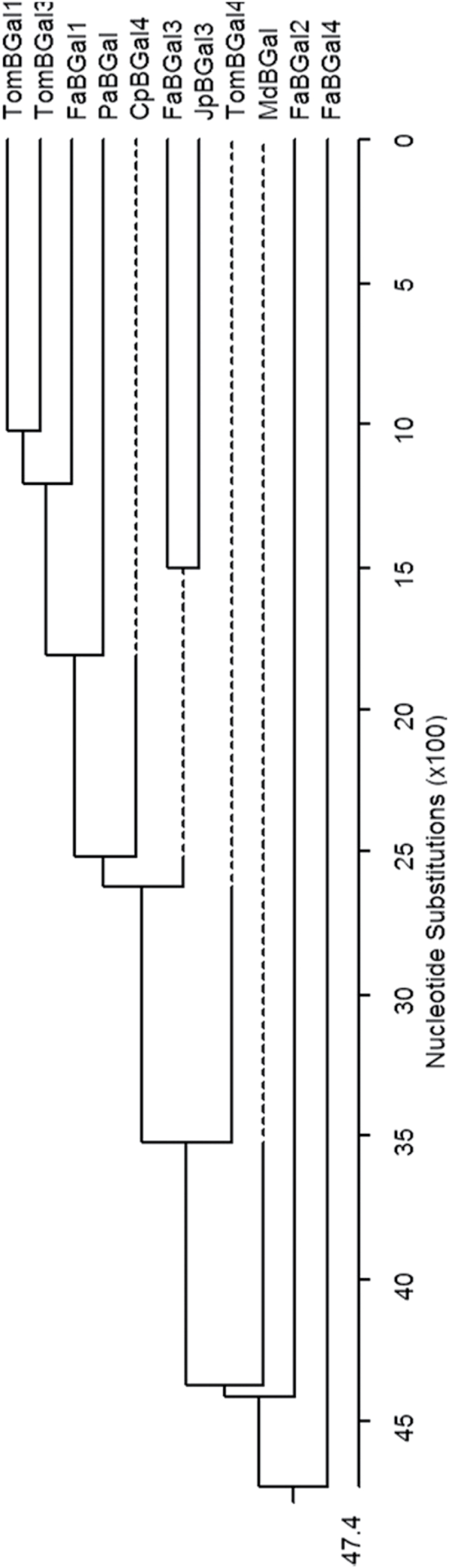
Phylogenetic analysis of selected fruit β-galactosidase proteins. The length of each pair of branches represents the distance between sequence pairs, while the units at the bottom of the tree indicate the number of substitution events. GenBank accession numbers and sources for the respective sequences are: *TomβGal4* (AF020390; *Solanum lycopersicum*); *CpβGal4* (AAC77377; *Carica papaya*); *FaβGal1* (AJ278703; *Fragaria* × *ananassa*); *FaβGal2* (AJ278704; *Fragaria* × *ananassa*); *FaβGal3* (AJ278705; *Fragaria* × *ananassa*); *FaβGal4* (KR189030; *Fragaria* × *ananassa*); *JpβGal3* (AB046543; *Pyrus pyrifolia*); *MdβGal* (L29451; *Malus domestica*); *PaβGal* (AB061017; *Persea americana*); *TomβGal1* (X83854; *Solanum lycopersicum*); and *TomβGal3* (CAA10173; *Solanum lycopersicum*). Sequences were aligned using MegAlign (Windows 32; MegAlign 5.0; DNASTAR)

A putative novel β-galactosidase gene, *FaβGal4*, was identified in the transcriptomic analysis of green versus red-ripened strawberry receptacles using a 35k microarray oligonucleotide-based platform. A BlastN search showed 99% similarity between the cDNA sequence of *FaβGal4* and *Fragaria vesca* β-galactosidase 16 mRNA (XM_004292664.2). The *FaβGal4* gene encodes a polypeptide of 909 amino acids that contains a glycosyl hydrolase family 35 domain and a galactose lectin binding domain. Phylogenetic tree analysis of the *FaβGal4* protein with other β-galactosidase fruit proteins from higher plants revealed the highest sequence identity with the *FaβGal2* protein from strawberry (84.5%) and low identity with *TomβGal1* (45.7%) and *TomβGal3* (47%), both from tomato, *PaβGal* (44.3%) from avocado (*Persea americana*), and *MdβGal* (47.2%) from apple (*Malus domestica*) (Fig. 1).

### Gene expression studies

**Fig. 2.**
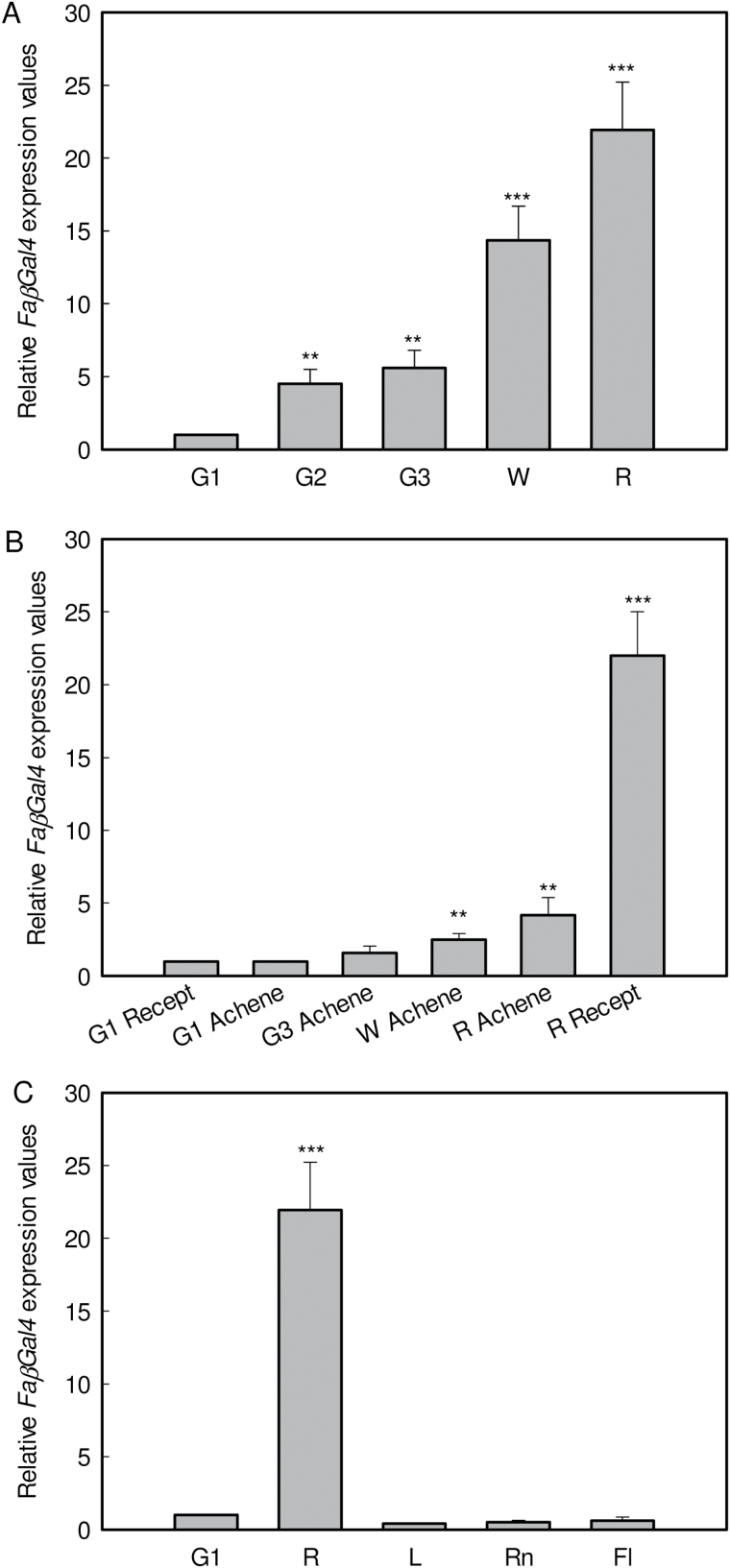
Expression of the strawberry *FaβGal4* gene during fruit receptacle development (**A**), achenes from fruits at different developmental stages (**B**), and in different vegetative tissues (**C**). Fl: flower; G1: small-sized green fruit; G2: medium-sized green fruits; G3: full-sized green fruit; L: leaf; R: red fruit; Rn: runner; W: white fruit;. Quantification was based on Ct values as described in Materials and Methods. The increase in the mRNA value was relative to the G1-Ct value, which was assigned an arbitrary value equal to unity. Mean values ± SD of five independent experiments are shown. In all figures, statistical significance with respect to the reference sample (G1 achenes in B and G1 fruit receptacle in A and C) was determined by the Student’s *t*-test. **P-value ˂ 0.01 and ***P-value ˂ 0.001.

Transcript analyses in fruits of the cultivated *Fragaria* × *ananassa*, cv. Camarosa, showed low expression of *FaβGal4* in the early stages of fruit development. There was a progressive increase in expression during the ripening process, which starts at the white stage of receptacle ripening, and the highest level of expression was reached in red fruit receptacles (Fig. 2A). An expression analysis of the other β-galactosidase genes previously described in strawberry, *FaβGal1–3*, showed that only *FaβGal4* was clearly related to the ripening process (see Supplementary Fig. 1 at *JXB* online).

*FaβGal4* transcript levels were very low in both achenes, independent of fruit ripening stage (Fig. 2B), and vegetative tissues (Fig. 2C) but increased dramatically in the ripe fruit receptacle, indicating the putative specificity of this gene in the fruit receptacle.

### *Hormonal regulation of* FaβGal4

**Fig. 3.**
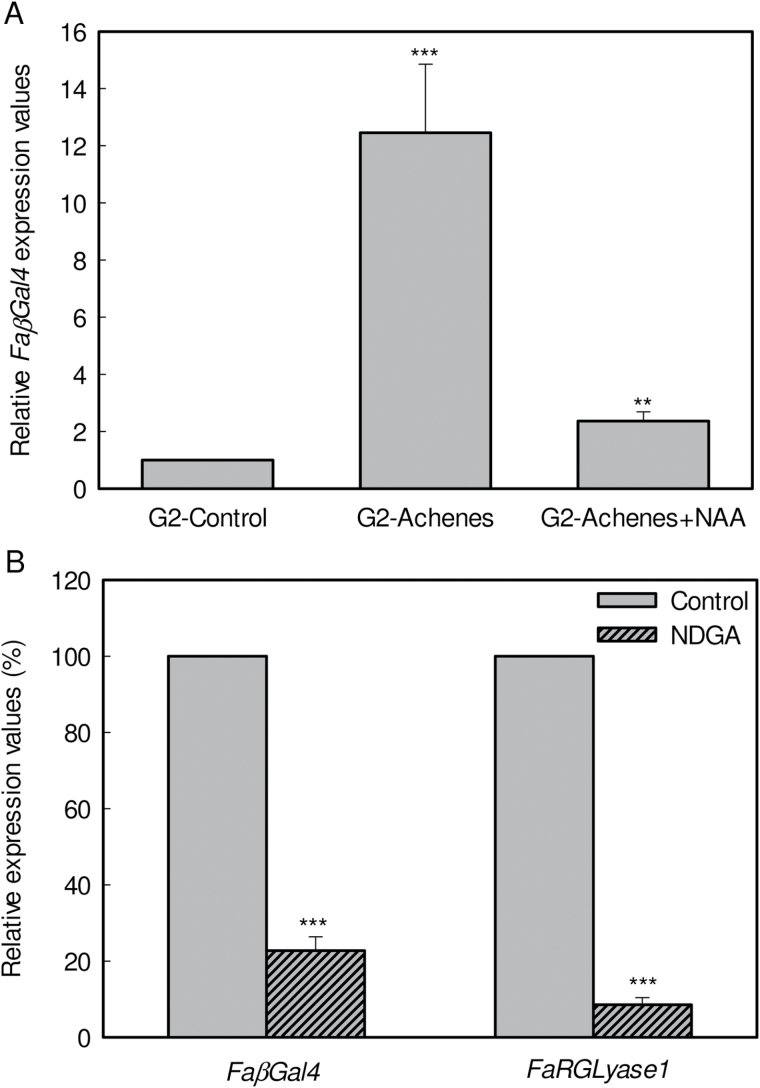
Hormonal regulation of *FaβGal4* expression. (**A**) Analysis of the effects of removing achenes from G2 developing fruits on *FaβGal4* expression estimated by qRT-PCR. The increase in mRNA values after achene removal and auxin treatment was relative to G2 fruit (control), which was assigned an arbitrary value equal to unity. G2-Achenes: G2 fruit receptacle without achenes; G2-Achenes+NAA: G2 fruit receptacle without achenes treated with 1mM NAA; Control: full-sized green fruit receptacle (G2-Control). (**B**) Analysis by qRT-PCR of *FaβGal4* and *FaRGLyase1* expression in strawberry fruits in response to NDGA. NDGA treatment performed for 8 days to G3-stage fruits. Control: G3 fruits injected with water; *FaRGLyase1*: rhamnogalacturonate lyase 1 (CO381780.1); NDGA: G3 fruits injected with NDGA (100 µM). The expression levels of both genes studied in NDGA-treated fruits are expressed as a percentage against their expression levels in control untreated fruits. Statistical significance with respect to the control samples was determined by the Student’s *t*-test. ***P-value ˂ 0.001 and **P-value < 0.01.

It has been proposed that the ABA to auxin content ratio in strawberry fruit receptacles constitutes a signal that triggers the fruit ripening process (Perkins-Veazie, 1995). Thus, we investigated whether *FaβGal4* expression was under the control of these two hormones. Because it is known that auxins present in strawberry receptacles are synthesized by the achenes, a comparative gene expression analysis was carried out between control and de-achened green fruits at the G2 stage that were or were not externally treated with NAA. A clear increase in the amount of *FaβGal4* transcript was detected in de-achened fruits (Fig. 3A). This increase did not occur, however, when de-achened fruits were treated with a lanolin paste containing NAA. These results clearly indicate that the expression of this gene was negatively regulated by auxins.

In addition, when the receptacle ABA content was depleted by adding NDGA, a significant reduction in the amount of *FaβGal4* and other cell wall enzyme transcripts, e.g. *FaRGLyase1*, was observed when compared against control fruits (Fig. 3B). This indicates that *FaβGal4* expression could be activated by ABA.

### *Phenotypic analysis of antisense* FaβGal4 *plants*

Nine independent kanamycin-resistant shoots carrying the antisense sequence of the *FaβGal4* gene were recovered, yielding an average transformation rate of 2%. The transgenic nature of the plants was confirmed by PCR amplification of a 220-bp fragment belonging to the *nptII* gene (results not shown). Transgenic *FaβGal4* plants showed a vegetative growth pattern similar to that of the control plants. However, during the first year of analysis, fruit yield was reduced by 60–80% in most of the lines compared with the control line. Furthermore, the proportion of malformed and small fruits in these lines was higher than in control. To estimate fruit quality, control and transgenic fruits were harvested at the stage of full ripening, and the weight, length, width, colour, soluble solids, and firmness were recorded. Results obtained during the first year of analysis are summarized in Table 1. Mean fruit weight and size were significantly lower in most transgenic lines when compared with control fruits, with the exception of lines β-Gal21 and β-Gal27. Interestingly, fruit colour, estimated by the L* a* b* colour space parameters, was significantly different in most transgenic lines. Transgenic fruits showed an increase on L* (lightness) and b* (yellowness), while a* (redness) was significantly increased only in lines β-Gal28 and β-Gal37. Soluble solids increased significantly in three of the transgenic lines, β-Gal25, β-Gal28, and β-Gal37. With respect to fruit firmness, lines β-Gal28 and β-Gal37 yielded fruits significantly firmer than control, with an increase in firmness of 47% and 31%, respectively. Based on this observation, these two lines were selected for further studies.

**Table 1.** Characteristics of ripened fruits in control and transgenic β-galactosidase plants. Fruits were harvested at the stage of full ripeness and data represent mean ± SD of a minimum of ten fruits per line, evaluated during the first year of analysis

Genotype	Weight (g)	Length (mm)	Width (mm)	Colour			Soluble solids (ºBx)	Firmness (N)
				L*	a*	b*		
Control	11.2±2.2	35.3±4.8	26.2±3.1	36.5±1.9	38.0±3.4	20.4±3.0	8.3±1.7	3.2±0.6
β-Gal15	9.5±1.8*	33.0±3.0	23.8±2.2*	38.7±3.3*	37.3±4.4	21.9±5.2	8.8±1.7	3.4±0.5
β-Gal18	7.8±1.6*	28.4±3.1*	24.4±2.2*	38.8±3.6*	39.5±5.1	21.3±3.3*	9.0±1.7	3.0±0.6
β-Gal19	7.8±1.8*	28.0±4.4*	24.0±2.6*	39.3±3.5*	37.8±3.6	22.3±3.5*	9.0±1.8	3.1±0.7
β-Gal21	10.2±3.1	32.2±5.4*	25.0±2.9	38.6±2.5*	37.9±3.3	23.1±3.9*	8.9±2.2	3.2±0.7
β-Gal24	7.3±2.1*	30.3±5.6*	22.9±3.4*	39.9±6.3*	39.8±5.5	26.0±5.5*	9.6±2.9	3.5±0.6
β-Gal25	5.2±2.0*	24.4±4.3*	22.6±3.1*	44.9±5.8*	40.7±4.7	28.1±4.2*	13.8±2.6*	3.5±0.9
β-Gal27	11.9±2.5	36.2±4.7	26.9±2.9	33.8±2.7*	36.9±3.2	18.1±3.5*	7.5±2.0*	3.4±0.6
β-Gal28	9.7±3.0*	28.8±4.1*	27.0±3.8	40.0±4.1*	43.2±3.8*	26.1±4.3*	10.0±2.7*	4.7±0.8*
β-Gal37	5.9±1.4*	22.7±3.8*	23.4±1.9*	40.5±3.2*	40.9±2.8*	25.8±3.2*	10.8±2.5*	4.2±0.9*

*Mann–Whitney *U* test shows significant difference from control at *P* = 0.05

Fruit quality parameters in selected transgenic lines were evaluated during two additional growing seasons, using each years’ daughter plants derived from vegetative propagation by runners. Results obtained in the two growing seasons were similar to those observed during the first year of analysis (Table 2). Transgenic fruits were smaller than control and showed higher soluble solid contents, especially those from line β-Gal37. Colour was also slightly modified in both transgenic lines. With respect to fruit firmness, both transgenic lines displayed significantly higher firmness values than control in the two years of analysis (Table 2): the average increase of firmness was 28%. Fruit yield was significantly reduced in both transgenic lines, with mean values of 179.2 ± 31.2, 63.7 ± 10.9, and 102.2 ± 29.2g of fruit per plant in control, non-transformed plants, and β-Gal28 and β-Gal37 transgenic lines, respectively. This decrease was due to a reduction in fruit weight but also a decrease in the number of fruits produced.

**Table 2.** Characteristics of ripened fruits in control and selected transgenic β-galactosidase plants. Fruits were harvested at the stage of full ripeness and data represent mean ± SD of a minimum of 50 fruits per line, evaluated during the second and third year of analysis. Mean separation within each year was performed by Tamhane T2 test at *P* = 0.05

	Genotype	Weight (g)	Length (mm)	Width (mm)	Colour			Soluble solids (ºBx)	Firmness (N)
					L*	a*	b*		
Second year	Control	12.2±3.8a	36.2±4.8a	27.5±3.8a	36.1±3.5b	38.4±3.7b	21.0±4.2a	6.9±1.5c	2.6±1.0b
	β-Gal28	8.2±2.6b	29.6±3.7b	25.8±2.8b	36.8±4.2b	40.2±4.7a	22.5±5.4b	7.9±1.3b	3.4±0.6a
	β-Gal37	6.3±1.5c	24.0±2.5c	23.8±2.2c	38.8±4.1a	38.7±4.4b	23.5±4.8b	8.9±1.7a	3.5±0.5a
Third year	Control	14.3±5.3a	39.5±6.2a	29.3±4.6a	35.5±2.2b	36.6±3.7a	19.5±3.5b	6.4±1.4c	3.6±0.8b
	β-Gal28	9.9±3.4b	34.7±5.8b	25.7±4.1b	38.9±4.2a	42.9±4.1b	25.42±5.1a	8.1±1.5b	4.6±1.0a
	β-Gal37	7.3±2.3c	26.0±3.9c	24.9±3.2b	40.1±4.5a	38.6±4.9c	24.3±3.8a	9.2±1.9a	4.4±0.8a

### FaβGal4 *gene expression and enzyme activity in transgenic fruits*

**Fig. 4.**
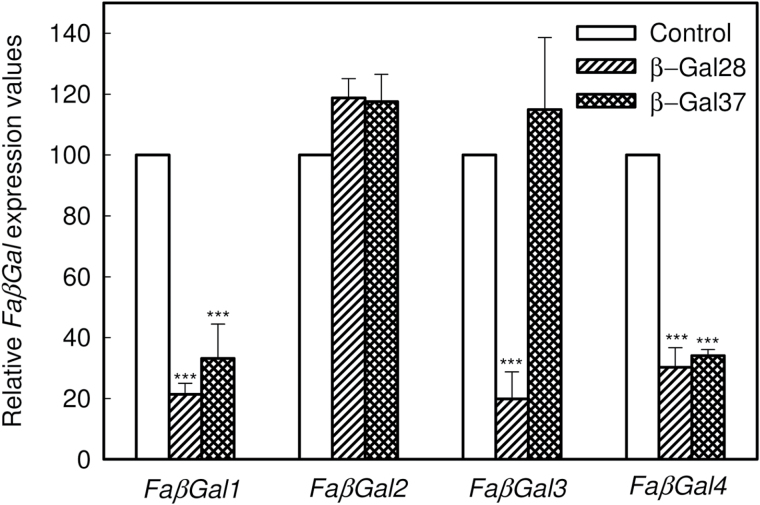
Relative *FaβGal4* expression, estimated by qRT-PCR, in ripe fruits from control and transgenic β-galactosidase lines. Bars represent mean ± SD of three independent RNA quantifications. Statistical significance with respect to the control line was determined by Dunnett’s multiple comparison test. ***P-value ˂ 0.001 and **P-value < 0.01.

The expression level of *FaβGal4*, as well as the other three β-galactosidase genes described in strawberry fruit (*FaβGal1*–*3*), was analysed in red fruits from control and selected β-Gal28 and β-Gal37 transgenic lines by qRT-PCR (Fig. 4). A significant down-regulation of *FaβGal4* was observed in ripe fruits from the two transgenic lines, with a similar level of silencing in the transgenic genotypes, close to 70%. Despite the *FaβGal4* sequence sharing a low similarity with the rest of the β-galactosidase genes analysed, both transgenic lines also showed a 70% decrease in the amount of *FaβGal1* mRNA levels (Fig. 4). Furthermore, β-Gal28 transgenic fruits showed an additional silencing of *FaβGal3*. By contrast, mRNA levels of *FaβGal2* were not modified in transgenic lines.

β-galactosidase enzymes are characterized by their ability to hydrolyse terminal, non-reducing β-galactosyl residues from numerous substrates. In this research, the term β-galactosidase activity refers to an enzyme that hydrolyses a β-galactosyl residue linked to a variety of aglycones, e.g. nitrophenyl-β-D-galactopyranoside (NPG), whereas exo-galactanase refers to an enzyme that is specific for the non-reducing end of galactan (Smith *et al.*, 2002). Both activities were measured in triplicate in protein extracts from ripe fruits of the two anti-*FaβGal4* selected lines. Total β-galactosidase activity was similar in control and transgenic fruits, 0.6±0.1 µmol NPG·g FW^−1^·h^−1^ in control versus 0.7±0.1 and 0.9±0.2 µmol NPG·g FW^−1^·h^−1^ in fruits from β-Gal28 and β-Gal37, respectively. Exo-galactanase activity, measured against a lupin galactan, was low, and no differences between control and transgenic lines were observed (1.1±0.5 µg·g FW^-1^·h^-1^ in control versus 0.8±0.1 and 2.3±1.3 µg·g FW^−1^·h^−1^ in fruits from β-Gal28 and β-Gal37, respectively).

### Cell wall analysis

The yield of CWM and soluble PAW fraction obtained per fresh weight of fruit was similar in control and transgenic lines, with average values of 0.93g CWM·100g FW^−1^ and 0.09g PAW·100g FW^−1^. CWM was sequentially fractionated with water, CDTA, Na_2_CO_3_, and KOH (1M and 4M) to extract fractions enriched in water soluble pectins, ionically bound pectins, covalently bound pectins, and hemicellulosic polymers, as described by Santiago-Doménech *et al.* (2008). The yields of the different cell wall fractions obtained were similar to those described by Santiago-Doménech *et al.* (2008) and Posé *et al.* (2013) in fruits of the same cv., with no significant differences observed between control and transgenic fruits in any of the fractions obtained (data not shown).

**Fig. 5.**
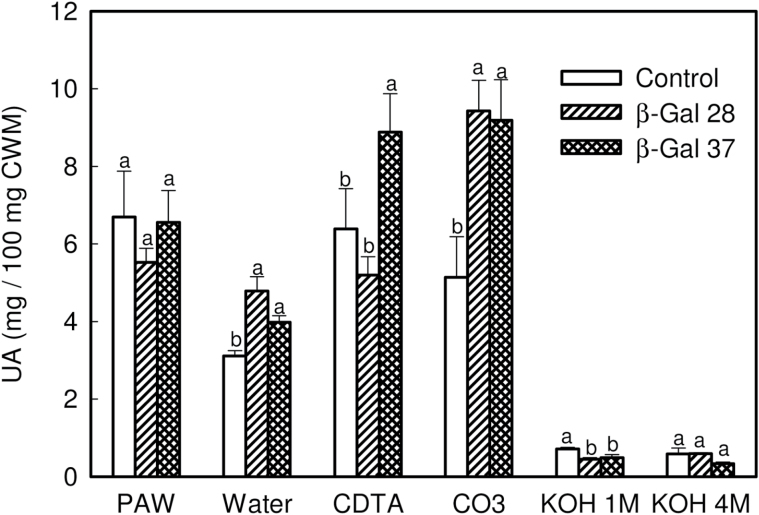
UA content, expressed as milligrams of UA per 100mg of CWM, in PAW and the different fractions isolated from cell walls from control and transgenic β-galactosidase ripe fruits. Bars represent mean ± SD of five independent measurements. Mean separation within each cell wall fraction by Dunnett’s multiple comparison test at *P* = 0.05.

**Fig. 6.**
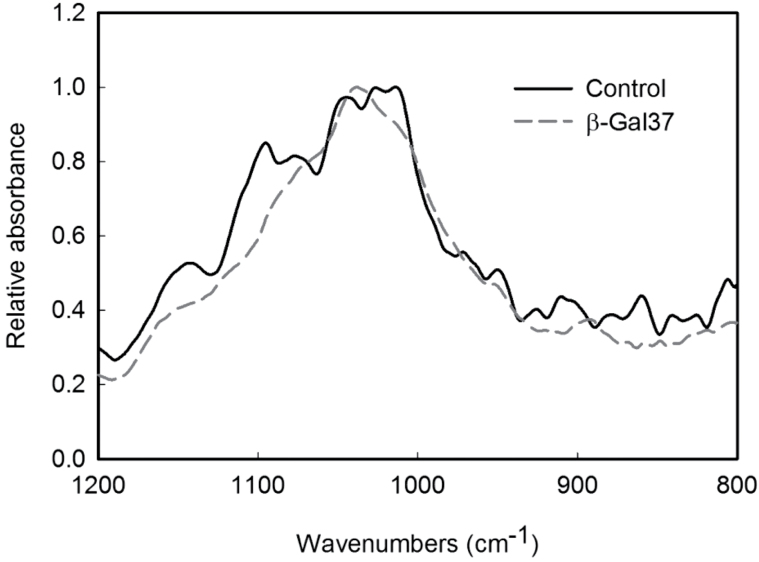
Attenuated total reflectance Fourier transform infrared spectroscopy absorbance spectra of the 1 M KOH fraction from control and transgenic β-Gal37 cell walls in the 1200–800cm^−1^ region.

UA content was measured in all fractions, and the results obtained are shown in Fig. 5. In both transgenic lines, the Na_2_CO_3_ fraction was enriched in UA when compared with control. A slight increase in UA was also observed in the transgenic water fractions. The amount of UA was also higher in the CDTA fraction from line β-Gal37, but not β-Gal28. The analysis of neutral sugars by gas chromatography revealed significant differences between the transgenic β-Gal37 line and control CWM. Transgenic CWM showed a 31% increase in Gal and Ara content (Table 3). The amounts of Rha and Fuc were also higher than control, although the differences were not statistically significant. By contrast, the amount of Xyl, Man, and Glc decreased slightly (Table 3). This general trend was observed in most cell wall fractions with some exceptions (Table 4). The control PAW fraction, which contained apoplastic free polymers solubilized by *in vivo* processes (Redgwell *et al.*, 1992), displayed the highest proportion of Rha, but this carbohydrate was not detected in transgenic PAW. Additionally, this fraction, as well as that of 4 M KOH, showed a slight increase in Glc when compared with control. Polymers soluble in CDTA from transgenic fruits showed a slight decrease in Fuc, Ara, Xyl, and Glc content. Similarly, Na_2_CO_3_-soluble pectins displayed large decreases in Fuc, Xyl, and Man. Regarding Gal content, all cell wall fractions from transgenic fruits contained a higher proportion of this carbohydrate (Table 4), with the greatest increase in the water and, especially, the 1 M KOH fractions (see Supplementary Fig. 2 at *JXB* online). Interestingly, the Gal increase in this last fraction was concomitant with large decreases in Xyl and Man and a high increase in Rha (Table 4). The modification of the carbohydrate composition in the 1 M KOH fraction as a result of *FaβGal4* silencing was confirmed by Fourier transform infrared spectroscopy (Fig. 6). Control fruits showed a spectral profile in the mid-infrared region at 1200–800cm^−1^, corresponding to the fingerprint region of carbohydrates, with peaks at 1014, 1026, 1045, 1077, and 1095cm^−1^. According to Kačuráková *et al.* (2000), homogalacturonan pectin profiles have maximum absorption bands at 1100 and 1017cm^−1^, RG-I shows the strongest peaks at about 1070 and 1043cm^−1^, and the main xyloglucan absorption band is at 1041cm^−1^. The 1 M KOH profile from control fruits could therefore be assigned to a mixture of RG-I and xyloglucans. The 1 M KOH profile from transgenic fruits was different, showing a main peak at 1038cm^−1^ and a shoulder at 1070cm^−1^. According to Kačuráková *et al.* (2000), these bands could be assigned to Ara and Gal, respectively, although the absorption infrared bands of other neutral sugars overlap in this region. This result suggests a change in the solubilization of RG-I present in the KOH fraction.

**Table 3.** Neutral sugar contents in CWM from ripe control and transgenic *FaβGal4* fruits. CWM was extracted from ripe fruits of control and antisense *FaβGal4* selected line β-Gal37 and the neutral sugar content was estimated by gas chromatography. Data represent mean values of three independent measurements. Different letters within columns indicate significant differences by Student’s *t*-test at *P* = 0.05

	Amount (mg.g CWM^-1^)						
	Rha	Fuc	Ara	Xyl	Man	Gal	Glc
Control	6.9a	5.7a	32.5b	48.4a	30.0a	78.5b	307.5a
β-Gal37	10.6a	8.2a	44.1a	45.5a	26.4a	103.4a	281.1a

**Table 4.** Neutral sugar composition in cell wall fractions from control and transgenic *FaβGal4* ripe fruits. CWM from ripe control and transgenic selected line β-Gal37 fruits was sequentially fractionated with water, CDTA, Na_2_CO_3_, and 1 M and 4 M KOH. The neutral sugar composition of the different cell wall fractions, the residue after cell wall fractionation, as well as the material soluble in PAW was estimated by gas chromatography and expressed in mol%. Data represent mean values of three independent measurements

	mol%	Rha	Fuc	Ara	Xyl	Man	Gal	Glc
PAW	Control	9.4	1.8	16.3	18.5	7.1	33.2	13.8
	β-Gal	0.0	2.3	21.6	20.0	5.6	34.9	15.6
H_2_0	Control	2.2	1.4	27.5	21.3	14.2	20.1	13.3
	β-Gal	6.0	1.8	25.2	13.8	9.1	37.0	7.2
CDTA	Control	4.8	2.0	32.2	9.9	9.5	35.5	6.2
	β-Gal	8.1	1.7	27.6	4.0	8.7	46.3	3.6
Na_2_CO_3_	Control	4.5	2.0	25.4	4.7	5.5	55.1	2.7
	β-Gal	6.3	0.9	27.5	1.9	2.0	59.4	2.1
1 M KOH	Control	1.6	2.1	15.2	47.6	7.7	12.9	13.0
	β-Gal	3.3	2.1	19.4	23.2	2.8	37.2	12.1
4 M KOH	Control	1.1	4.0	12.1	32.4	6.3	18.5	25.7
	β-Gal	1.8	4.5	10.2	28.1	3.9	21.3	30.3
CWM residue	Control	0.7	0.2	1.8	3.3	5.6	3.0	85.5
	β-Gal	0.6	0.2	2.4	3.3	3.5	2.2	87.9

## Discussion

A common feature of the cell wall disassembly process taking place during ripening of fleshy fruits is the loss of galactose from pectin side chains, supposedly due to the action of β-galactosidase enzymes (Smith and Gross, 2000; Brumell, 2006). In strawberry, Trainotti *et al.* (2001) identified three β-galactosidase genes. All of them were expressed in young expanding leaves, stolons, flowers, and green fruits, but only *FaβGal1* showed increased expression during fruit ripening. Our comparative transcriptomic analysis between strawberry receptacles from immature (green) and ripened (red) strawberry fruits showed a sequence encoding a novel putative β-galactosidase gene, *FaβGal4*, whose expression was strongly up-regulated, suggesting that this gene could play a pivotal role in strawberry fruit cell wall degradation and fruit softening.

Bioinformatic analyses showed a low identity between *FaβGal4* full-length cDNA, or its orthologue in *F. vesca*, and the three β-galactosidase genes previously described in strawberry. At the protein level, the predicted *FaβGal4* shares the highest identity with *FaβGal2*. The deduced protein of *FaβGal4* contains the consensus sequence pattern of the putative active site characteristics of the glycosyl hydrolase family 35 (Henrissat *et al.*, 1995), and, interestingly, it is identical to the putative active site for the other three β-galactosidase genes described in strawberry (Trainotti *et al.*, 2001). These three genes showed β-galactosidase activity when expressed *in vitro* (Trainotti *et al.*, 2001) and, therefore, it is likely that the *FaβGal4* product also has β-galactosidase activity. However, enzymatic activity assays should be performed in order to definitively address this question.

### FaβGal4 *is mainly expressed in ripe fruit receptacles and is regulated by auxin and ABA*

The expression pattern of *FaβGal4* during receptacle fruit growth and maturation was clearly ripening-related, with maximum gene expression in red ripe fruits. This expression pattern was similar to those previously reported for other strawberry genes encoding pectin-degrading enzymes, e.g. pectate lyases, polygalacturonases, or rhamnogalacturonate lyase (Medina-Escobar *et al.*, 1997; Redondo-Nevado *et al.*, 2001; Benitez-Burraco *et al.*, 2003; Quesada *et al.*, 2009; Molina-Hidalgo *et al.*, 2013).

During the development of strawberry fruit, the level of auxins produced by achenes and released into the receptacle declines, which induces the ripening process (Perkins-Veazie, 1995). Several studies have shown that the removing the achenes from the fruit surface induces the expression of many strawberry fruit ripening-related genes that encode cell wall enzymes (Medina-Escobar *et al.*, 1997; Redondo-Nevado *et al.*, 2001; Benitez-Burraco *et al.*, 2003). This was also the case for *FaβGal4*. More recently, Chai *et al.* (2011) and Jia *et al.* (2011) provided molecular evidence indicating that ABA is a signal molecule that, at least, can promote the strawberry ripening-related production of anthocyanins. In fact, ABA levels gradually increase concomitant with the ripening process in strawberry fruits (Chai *et al.*, 2011; Jia *et al.*, 2011). When strawberry fruits were treated at the green stage with NDGA, an inhibitor that blocks the synthesis of ABA, *FaβGal4* was significantly down-regulated, as has been observed for other cell wall enzymes (Molina-Hidalgo *et al.*, 2013). Altogether, these results indicate a putative co-regulation of *FaβGal4* with genes encoding other pectin-degrading enzymes, and support a physiological role related with the enzymatic degradation of the cell wall in ripe fruits leading to fruit softening.

### *Antisense down-regulation of* FaβGal4 *reduces strawberry fruit softening*

Transgenic strawberry plants carrying a 300-bp antisense sequence of *FaβGal4* under the control of the constitutive promoter CaMV35S were generated to get insight into the role of this gene on fruit softening and cell wall disassembly. In general, fruit yield and fruit weight were reduced in most transgenic lines, as has been observed in other transgenic lines with pectinases down-regulated, e.g. pectate lyase transgenic plants (Jiménez-Bermúdez *et al.*, 2002; Youssef *et al.*, 2009). Average fruit size and weight generally decrease when micropropagated strawberry plants are used directly for fruit production, but this side effect of the *in vitro* tissue culture phase disappears when the progeny of micropropagated plants is evaluated (Cameron *et al.*, 1989; López-Aranda *et al.*, 1994). In *FaβGal4* transgenic plants, the reduction of fruit weight was stably maintained after three rounds of runner propagation in the greenhouse, suggesting that this effect was due to *FaβGal4* silencing rather than the *in vitro* micropropagation of plants. In strawberry, fruit size depends mainly on the number of achenes per fruit, the flower position, and the receptacle sensitivity to auxin (Perkins-Veazie, 1995). In the case of the two transgenic lines selected, a marked reduction of the average number of achenes per fruit was observed, with mean values of 240.1 in control plants, 105.6 in β-Gal28, and 69.5 in β-Gal37. However, the mean number of achenes per gram of fruit was only statistically different to the control in the case of β-Gal28 fruits. These results suggest that the decrease in fruit size was mainly due to deficient fertilization in transgenic plants as a result of *FaβGal4* silencing. Early and late male gametophyte development genes with high homology to β-galactosidases have been identified in pollen from tobacco, *Arabidopsis*, and rice (Tursun *et al.*, 2003; Hrubá *et al.*, 2005). The products of these genes may participate in cell wall loosening during young pollen expansion after microspore mitosis and/or may be associated with pollen germination and pollen tube penetration through the style (Hrubá *et al.*, 2005).

Two out of the nine transgenic lines obtained produced fruit significantly firmer than control at the ripe stage. The firmer fruit phenotype was stably maintained during three growing seasons. Transgenic fruits of selected lines were on average 30% firmer than control, an increase in firmness similar to that obtained when down-regulating a pectate lyase (Jiménez-Bermúdez *et al.*, 2002) or a polygalacturonase gene (Quesada *et al.*, 2009). The increase in fruit firmness cannot be ascribed to the reduction in fresh weight, because no correlation between fruit weight and firmness was observed among the different transgenic lines obtained. Transcriptomic analysis of transgenic fruit from the selected lines showed a significant reduction, 70%, of *FaβGal4* mRNA levels when compared with control fruit. Although *FaβGal4* did not share significant sequence homology with the other three β-galactosidase genes previously described in strawberry, the constitutive expression of an antisense *FaβGal4* sequence also reduced *FaβGal1* mRNA amounts in the two selected lines, as well as *FaβGal3* in fruits from β-Gal28. By contrast, *FaβGal2* expression was not modified in transgenic fruits. These results indicate an intricate regulation of β-galactosidase genes during the ripening of strawberry fruit. Additionally, despite the high reduction in the expression of β-galactosidase genes, neither β-galactosidase activity nor exo-galactanase activity were reduced in transgenic ripe fruit. Previous studies in transgenic tomato showed a complex relationship between the expression of the different β-galactosidase genes involved in fruit ripening and β-galactosidase activity. Antisense *TBG4* tomato plants showed a 40% reduction in fruit softening although *TBG4* silencing was only observed at the mature green stage (Smith *et al.*, 2002). The expression of *TBG3* was also altered in those antisense tomato fruit, down-regulating or up-regulating its expression depending on the ripening stage. Moctezuma *et al.* (2003) found an altered expression of *TBG4* and *TBG5* in tomato fruits with *TBG6* down-regulated by antisense transformation. Contrary to the report of Smith *et al.* (2002), the suppression of *TBG6* was observed during the whole ripening process. Although transgenic *TBG6* fruits had no differences in softening, increased fruit cracking and cuticle thickness, and a reduced locular space were observed (Moctezuma *et al.*, 2003). As regards β-galactosidase activity, neither Smith *et al.* (2002) nor Carey *et al.* (2001) found a decrease in total β-galactosidase activity in transgenic antisense *TBG4* or *TBG1* fruit, respectively. However, the silencing of *TBG4* reduced exo-galactanase activity, measured against a lupin galactan, at the breaker plus 3 days developmental stage, returning to wild-type control levels at breaker plus 7 days stage (Smith *et al.*, 2002). The scenario was more complex when modifying the expression levels of *TBG6* (Moctezuma *et al.*, 2003). In these antisense fruits, β-galactosidase activity was significantly higher than in control at early stages of development, returning to wild-type levels at the later stages. However, exo-galactanase activity was reduced at the breaker stage (Moctezuma *et al.*, 2003). These results could be explained by the large number of β-galactosidase isoforms with different hydrolysing abilities against the native cell wall polysaccharides and synthetic products that are expressed in fruit (Tateishi, 2008). The *in vitro* expression of *FaβGal4* needs to be investigated to clarify the actual substrate of this protein.

### *Antisense* FaβGal4 *down-regulation increases cell wall galactose levels and reduces pectin solubilization*

According to Redgwell *et al.* (1997), Gal levels decrease 18% in ripe strawberry fruit when compared with green immature fruit. The silencing of *FaβGal4* increased Gal by 30% in transgenic ripe fruit, but also induced a significant increase in the Ara content. After cell wall fractionation, it was observed that all fractions contained higher amounts of Gal than control, with the largest increases present in the H_2_O, CDTA, and especially the 1 M KOH fractions. KOH-soluble fractions comprise mainly hemicellulosic polymers, but also contain pectins (Koh and Melton, 2002; Santiago-Doménech *et al.*, 2008; Posé *et al.*, 2013). Interestingly, Redgwell *et al.* (1997) found that the highest Gal loss, 82.3% of total cell wall Gal, occurred in the KOH fraction and the CWM residue, while only 17.7% was lost from the CDTA/Na_2_CO_3_ polyuronide-enriched fractions. This trend was observed in most of the fruit analysed, including species where an increase of β-galactosidase activity during fruit ripening had previously been reported, e.g. tomato, kiwifruit, and avocado (Redgwell *et al.*, 1997). The high Gal content in the 1 M KOH fraction from antisense *FaβGal4* fruits suggests that Gal loss takes place mostly in arabinogalactan pectins loosely bound to xyloglucans by covalent cross-links and/or physically entangled within the matrix glycan. It is noteworthy that this is the first report of the modification of cell wall Gal levels in fruit by suppression of a β-galactosidase gene. Total cell wall Gal content was not modified in antisense *TBG4* tomato fruit, although decreased free galactose levels were observed in transgenic fruit at mature green stage, returning to wild-type levels during ripening (Smith *et al.*, 2002).

The relationships among loss of neutral sugars, pectin solubilization, and fruit firmness are far from clear. Some fruits such as apple and nashi pear showed a marked loss of Gal but pectin solubilization was slight (Redgwell *et al.*, 1997). Conversely, plum did not show any Ara/Gal loss but showed extensive pectin depolymerization (Redgwell *et al.*, 1997). In *FaβGal4*-silenced fruits, the increase of cell wall levels of Gal was paralleled by a reduction in polyuronide solubilization, reflected in the higher amount of Na_2_CO_3_-soluble pectins. Additionally, a preliminary analysis of these pectins by atomic force microscopy showed larger polymers in transgenic samples than in control samples, suggesting a reduced depolymerization during ripening (unpublished results). Previous studies in transgenic strawberry plants with pectate lyase or polygalacturonase genes silenced demonstrated that the increased firmness in these transgenic fruits was a result of reduced pectin solubilization and depolymerization (Santiago-Doménech *et al.*, 2008; Posé *et al.*, 2013; Posé *et al.*, 2015). In both cases, cell walls from transgenic fruit displayed higher amounts of ionically and covalently bound pectins, those soluble in CDTA and Na_2_CO_3_, respectively, than wild type. Whether the reduction in fruit softening in transgenic *FaβGal4* fruits is directly due to the increase of cell wall Gal content or by an indirect effect on pectin solubilization needs to be elucidated. The reduced loss of Gal from hairy regions of pectins could limit the access of other cell wall-modifying proteins, such as polygalacturonase or pectate lyase, to their substrate, therefore limiting pectin solubilization and, likely, depolymerization. As an alternative hypothesis, galactosyl residues could have a direct effect on the mechanical properties of the cell wall. In pea (*Pisum sativum*) cotyledons, the increased pectin galactan content at late developmental stages correlated with an increase in firmness (McCartney *et al.*, 2000). The *Arabidopsis* mutant *MUR3* lacks a xyloglucan-specific galactosyl transferase and its cell walls exhibit reduced xyloglucan galactosidation (Peña *et al.*, 2004). This modification markedly reduces cell wall strength during hypocotyl growth, although this effect could be related to a modulation of xyloglucan endo-transglycosidase/hydrolase activity (Peña *et al.*, 2004). The ectopic expression of a fungal endo-β-galactanase in potato tuber reduces RG-I linear β-1,4-galactan and also modifies the physical properties of the tissue, making it more brittle when subjected to compression (Ulvskov *et al.*, 2005). A putative role of *FaβGal4* on the release of galactosyl-containing oligosaccharides that trigger the ripening process also cannot be discarded. Gross (1985) found that mature green tomato fruit infiltrated with free Gal produced ethylene and ripened earlier than control fruits. More recently, Lahaye *et al.* (2013) found indirect evidence for the role of Gal on fruit ripening; they observed positive correlations between some firmness quantitative trait loci and cell wall Gal content, galactosylated pectins, and galactosylated xyloglucans in tomato. This hypothesis could explain the pleiotropic effect of *FaβGal4* silencing on fruit colour and on the expression of the other β-galactosidase genes.

### Conclusions

The results obtained support a key role for *FaβGal4* in the softening of strawberry fruit. Transgenic fruits with *FaβGal4* silenced showed a 30% increase in fruit firmness at the ripe stage. At the cell wall level, these transgenic fruits contained more Gal in all cell wall fractions than wild type, but also displayed lower polyuronide solubilization. Although the removal of Gal from cell walls is a general feature of the ripening process in many fruits, its relationship with pectin solubilization and fruit firmness is controversial. Our results shed light on these processes and clearly indicate a close connection between Gal levels and polyuronide solubilization. The reduction of Gal loss from galactosyl-containing side chains in the wall might result in decreased porosity, obstructing the access of other cell wall enzymes to their substrates, leading to reduced softening. Alternatively, the increased level of galactosyl residues could have a direct effect on the mechanical properties of the cell wall. This work also indicates that *FaβGal4* is a good candidate for the biotechnological or conventional improvement of strawberry texture.

## Supplementary material

Supplementary data are available at *JXB* online

Supplementary Figure 1: Relative expression of the different β-galactosidase genes during fruit receptacle development, estimated by qRT-PCR. Bars represent mean ± SD of three independent RNA quantifications

Supplementary Figure 2: Increase of Gal content, expressed as a percentage of control fruits, in CWM, PAW, and the different cell wall fractions isolated from transgenic β-Gal37 ripe fruits.

Supplementary Data
