# Supplementary material for: Antisense down-regulation of the strawberry β-galactosidase gene FaβGal4 increases cell wall galactose levels and reduces fruit softening
Source: J Exp Bot. 2015 Nov 19;67(3):619–31. doi: 10.1093/jxb/erv462 (PMC4737064; doi:10.1093/jxb/erv462)
Supplement: Supplementary Data [file supp_67_3_619__index.html]

Antisense down-regulation of the strawberry β-galactosidase gene FaβGal4 increases cell wall galactose levels and reduces fruit softening — Antisense down-regulation of the strawberry β-galactosidase gene FaβGal4 increases cell wall galactose levels and reduces fruit softening — Supplementary Data 

# Antisense down-regulation of the strawberry β-galactosidase gene *FaβGal4* increases cell wall galactose levels and reduces fruit softening

## Supplementary Data

Data files

- Supplementary Data - Supplementary Data
