# Supplementary material for: Antisense down-regulation of the strawberry β-galactosidase gene FaβGal4 increases cell wall galactose levels and reduces fruit softening
Source: J Exp Bot. 2015 Nov 19;67(3):619–31. doi: 10.1093/jxb/erv462 (PMC4737064; doi:10.1093/jxb/erv462)

Antisense down-regulation of the strawberry β-galactosidase gene *FaβGal4* increases cell wall galactose levels and reduces fruit softening

Candelas Paniagua[1], Rosario Blanco-Portales[2], Marta Barceló-Muñoz[3], Juan A. García-Gago[1], Keith W. Waldron[4], Miguel A. Quesada[5], Juan Muñoz-Blanco[2], José A. Mercado[1]

Supplementary Figure 1: Relative expression of the different βGal genes during fruit receptacle development, estimated by qRT-PCR. Bars represent mean±SD of three independent RNA quantifications

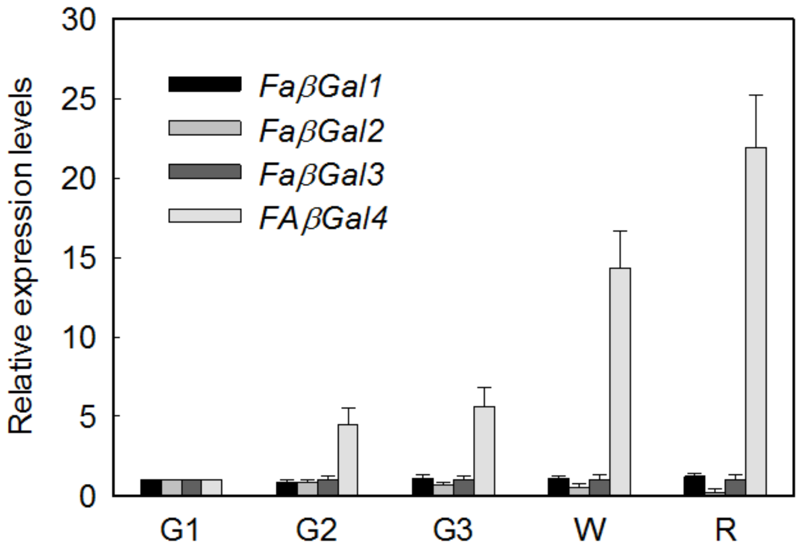

Supplementary Figure 2: Increase on Gal content, expressed as percentage of control fruits, in CWM, PAW and the different cell wall fractions isolated from transgenic β-Gal37 ripe fruits.

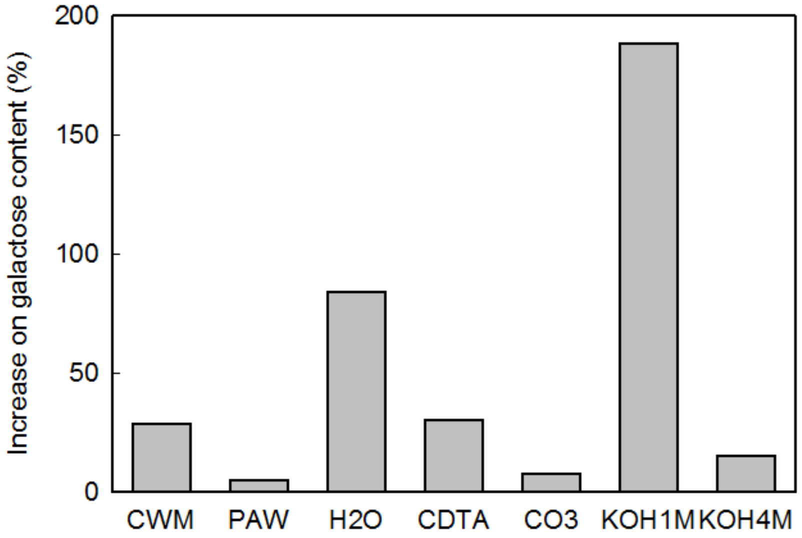

Supplement: Supplementary Data [file supp_erv462_Supplementary_figures.pdf]
